# Robot-assisted radical nephroureterectomy for right renal pelvic tumor post sacrocolpopexy with mesh: A case report

**DOI:** 10.1016/j.ijscr.2024.109523

**Published:** 2024-03-15

**Authors:** Koki Sugimura, Satoru Kira, Hiroshi Shimura, Takanori Mochizuki, Norifumi Sawada, Takahiko Mitsui

**Affiliations:** a Department of Urology, Kameda Medical Center, Chiba, Japan; b Department of Urology, University of Yamanashi Graduate School of Medical Sciences, Chuo, Yamanashi, Japan

**Keywords:** Radical nephroureterectomy, Pelvic organ prolapse, Mesh, Robotic surgery

## Abstract

**Introduction:**

Robot-assisted nephroureterectomy for upper tract urothelial carcinoma has been increasingly performed as a minimally invasive procedure recently. However, there are concerns regarding its adoption in highly complex cases with dense adhesions.

**Presentation of case:**

An 86-year-old woman presented to our hospital with gross hematuria one year after having undergone robot-assisted sacrocolpopexy using a mesh for pelvic organ prolapse. Cystoscopy revealed hematuria from the right ureteral orifice. Computed tomography suggested right hydronephrosis; retrograde pyelography showed a defect in the right renal pelvis with class V urine cytology of the urine from the right kidney. Based on these findings, a right renal pelvic tumor was diagnosed, and robot-assisted nephroureterectomy was performed. The patient was discharged on postoperative day 7 without complications.

**Discussion:**

To the best of our knowledge, this is the first case report in which robot-assisted radical nephroureterectomy was performed after robot-assisted sacrocolpopexy with a mesh. Dense tissue adhesions are encountered not only between the bladder and the anterior vaginal wall but also around the right ureter in the pelvis. In this case, dense adhesions were confirmed around the right ureter in the pelvis.

**Conclusion:**

Robot-assisted nephroureterectomy may be considered an option for minimally invasive surgery in cases with a history of pelvic organ prolapse surgery using mesh.

## Highlights

• This is the first case report wherein RANU was performed after RSC with a mesh.
• Dense tissue adhesions can appear around the right ureter in the pelvis.
• RANU may be considered for cases with a history of POP surgery using mesh.

## 1 Introduction

Following the successful visualization in three-dimensional high-resolution and ease of mobility by the da Vinci robotic system, robot-assisted radical nephroureterectomy (RANU) for upper tract urothelial carcinoma is increasingly being used as a minimally invasive procedure [1]. Compared to laparoscopic or open nephroureterectomy, RANU has several merits including lesser blood loss, pain, and hospital stay [1]. However, incorporating it in highly complex cases with dense adhesions remains controversial.

Here, we report a case of RANU for a right renal pelvic tumor with dense mesh adhesion after robot-assisted sacrocolpopexy (RSC).

The work has been reported in line with the SCARE criteria [2].

## 2 Presentation of case

**Fig. 1:**
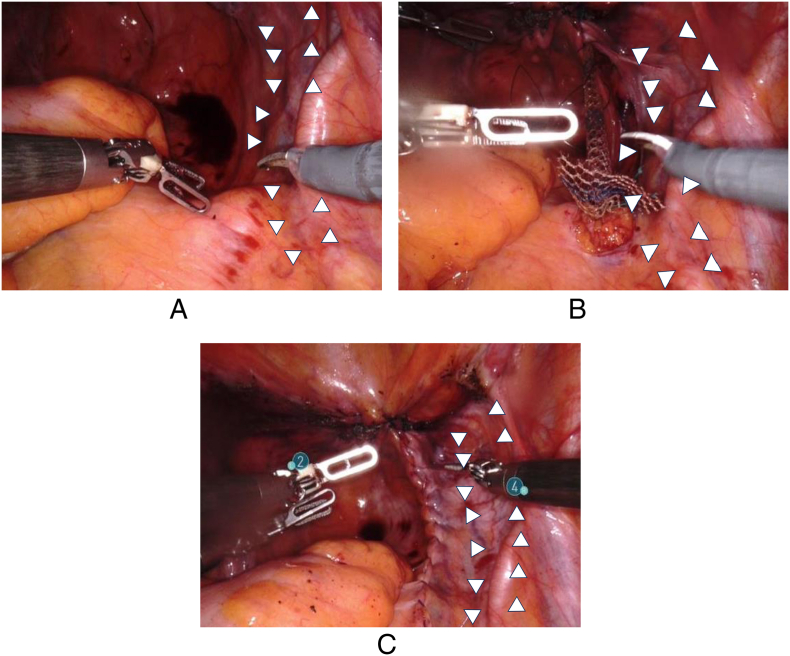
Intraoperative findings. (A): Right ureter (white arrows) in pelvis before insertion of mesh. (B): Right ureter (white arrows) in pelvis under insertion of mesh. (C): Right ureter (white arrows) in pelvis after insertion of mesh.

**Fig. 2:**
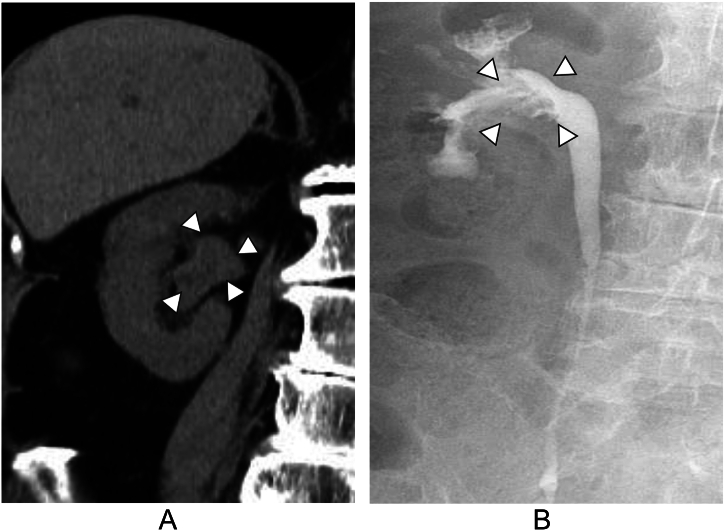
Abdominal computed tomography scan and retrograde pyelography images. (A): Abdominal computed tomography scan shows right hydronephrosis (white arrows). (B): Retrograde pyelography shows defect (white arrows) of contrast agent in the right pelvis.

**Fig. 3:**
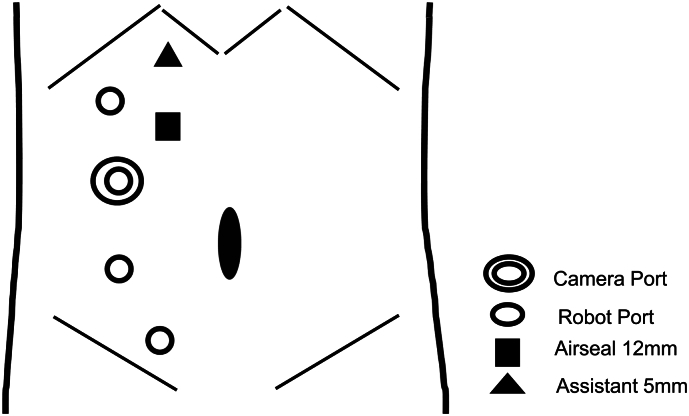
Schema of trocar placement. White circles indicate 8 mm robotic ports, double circles indicate camera ports, black squares indicate 12 mm assistant ports, and black triangles indicate 5 mm assistant ports for river elevation.

**Fig. 4:**
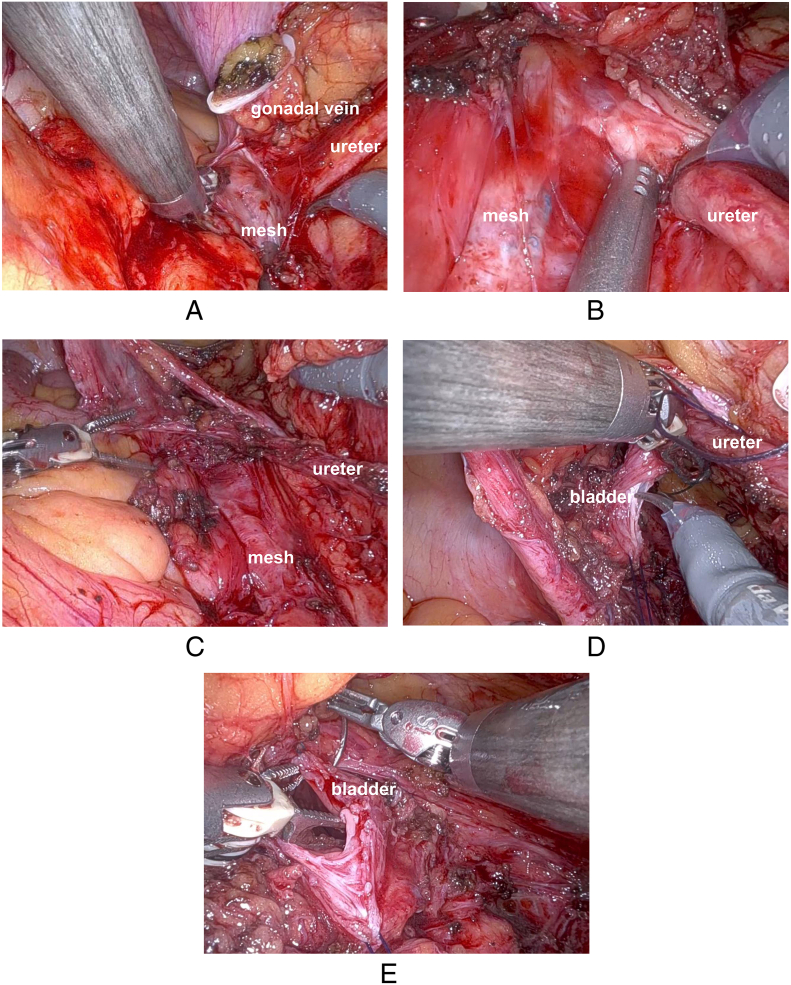
Images showing the adhesion between ureter and mesh during RANU. (A): Adhesion between ureter and mesh. (B): Magnified view of adhesion between ureter and mesh. (C): Isolation of ureter from mesh. (D): Bladder cuff excision. (E): Closure of bladder cuff.

An 86-year-old woman had undergone RSC using a mesh for pelvic organ prolapse (POP) at our department. (Fig. 1A-C) One year after surgery, she presented to our hospital with a chief complaint of gross hematuria. Cystoscopy revealed hematuria spouting from the right ureteral orifice. Computed tomography suggested right hydronephrosis, and retrograde pyelography showed a defect in the right renal pelvis with class V urine cytology of the urine from the right kidney (Fig. 2A, B). Based on these findings, we made a diagnosis of right renal pelvic tumor, and decided to perform RANU. The patient was placed in the flank position and flexed. Four 8 mm robotic trocars, and 12 mm and 5 mm assistant trocars were inserted (Fig. 3). We performed RANU using the Da Vinci X (Intuitive Surgical, Sunnyvale, CA, USA). Although the nephrectomy procedure progressed smoothly, ureterectomy in the pelvis was affected by dense adhesions due to the mesh being placed by the RSC (Fig. 4A-C). After ureter was clipped and mobilized into the bladder without urine leakage, bladder cuff excision and closure of bladder cuff with suture in two layers were performed. (Fig. 4D, E) Finally, RANU was safely completed without lymph node dissection and drain placement. The operative and console times were 277 min and 196 min, respectively, with an estimated blood loss of 93 mL. The patient was discharged on postoperative day 7 with no complications. Histopathology revealed a noninvasive high-grade papillary urothelial carcinoma in the renal pelvis with a T stage of pTa and negative surgical margins including the part of bladder cuff.

## 3 Discussion

To our knowledge, this is the first report of a case in which RANU was performed after RSC with a mesh. On characteristics of the procedure, mesh used in the RSC was placed close to the right ureter in the pelvis to fix the proximal parts of the mesh to the right side of the sacral promontory. To create a tunnel for the mesh placement, the parietal peritoneum was dissected from the sacral promontory to the anterior vaginal wall. Thus, dense tissue adhesions are encountered not only between the bladder and the anterior vaginal wall but also around the right ureter in the pelvis. In this case, dense adhesions were confirmed around the right ureter in the pelvis. However, we successfully performed a nephroureterectomy in this complex case using robotic technology.

Similar to the situation in this case, Robot-assisted radical prostatectomy (RARP) in patients with a history of prior inguinal hernia repair with mesh can be supposed [3]. It has been suggested that a history of inguinal hernia repair with mesh makes RARP more difficult because of dense adhesion induced by mesh insertion [3]. In our case, we chose the transperitoneal approach as we considered an extraperitoneal approach to be more difficult owing to retroperitonealization of the meshes during RSC. Although it is unclear which approach is better, in the case of RARP in patients with a history of prior inguinal hernia repair with mesh, there were no differences in either of the approaches [3].

Currently, the peak age for POP is 70, and it is estimated to gradually increase over the next 20–30 years [4]. Owing to the benefit of minimally invasive surgery for older patients, RSC with mesh has been growing for the management of POP [5]. However, the diagnosis of urothelial carcinoma reaches the peak age during 70–79 years similar to POP [6]. Although cases of urothelial carcinoma with history of POP surgery with mesh might increase, RANU could be considered an option for minimally invasive surgery in this situation.

## 4 Conclusion

We successfully performed RANU with a prior history of POP surgery using a mesh. RANU may be considered an option for minimally invasive surgery in such complex cases.

## Consent

We obtained consent from the patient for publication of this CASE report. A copy of the written consent is available for review by the Editor–in–Chief of this journal on request.

## Ethical approval

Ethics approval is not required for case reports deemed not to constitute research at the IRB/Ethics Committee of University of Yamanashi Hospital.

## Funding

This study did not receive any specific grants from funding agencies in the public, commercial, or nonprofit sectors.

## Author contribution

Koki Sugimura; Conceptualization, Investigation, and Writing Original draft preparation,

Satoru Kira*; Writing, Reviewing, Editing, and Supervision,

Hiroshi Shimura; Validation and Data curation,

Takanori Mochizuki; Validation and Data curation,

Norifumi Sawada; Validation, Visualization, and Supervision,

Takahiko Mitsui; Editing and Supervision.

## Guarantor

Takahiko Mitsui.

## Research registration number

Not applicable.

## Conflict of interest statement

None declared.
